# Supplementary material for: Stem Cell Factors BAM1 and WOX1 Suppressing Longitudinal Cell Division of Margin Cells Evoked by Low-Concentration Auxin in Young Cotyledon of Arabidopsis
Source: Int J Mol Sci. 2025 May 15;26(10):4724. doi: 10.3390/ijms26104724 (PMC12120846; doi:10.3390/ijms26104724)
Supplement: Supplementary file 1 [file ijms-26-04724-s001.zip › ijms-3583564-supplementary.pdf]

Supplementary Information

Text summary:

Figure S1 BAM1 regulates margin cell development specifically

Figure S2 BAM1 and WOX1 expression pattern in young cotyledon.

Figure S3 mPS-PI stained mature embryos of *bam1bam2bam3*

Table S1 The sequence of primers used in genotyping and qRT-PCR

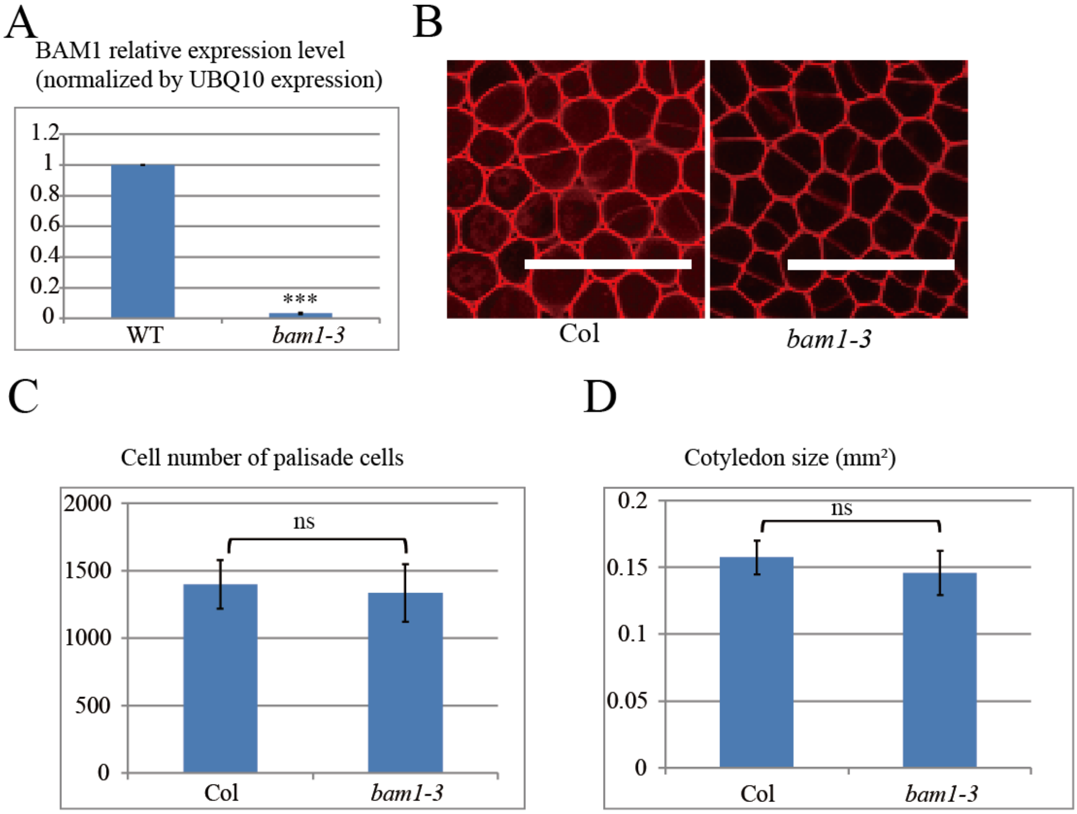

Figure S1 BAM1 regulates margin cell~~s~~ development specifically

(A) The quantitative RT-PCR detection of BAM1 transcripts in Col and *bam1-3*. Data are presented as mean ± SD for five independent experiments. *** $p < 0.001$.

(B) Palisade cells in Col (left panel) and *bam1-3* (right panel) at 2 dag. Bars = 50 µm.

(C) Quantification of the total cell number of palisade cells in Col and *bam1-3* at 2 dag. Data are presented as mean ± SD (Student's *t*-test. ns, no significant difference, n = 10)

(D) Quantification of the cotyledon size in Col and *bam1-3* at 2 dag. Data are presented as mean ± SD (Student's *t*-test. ns, no significant difference, n = 10).

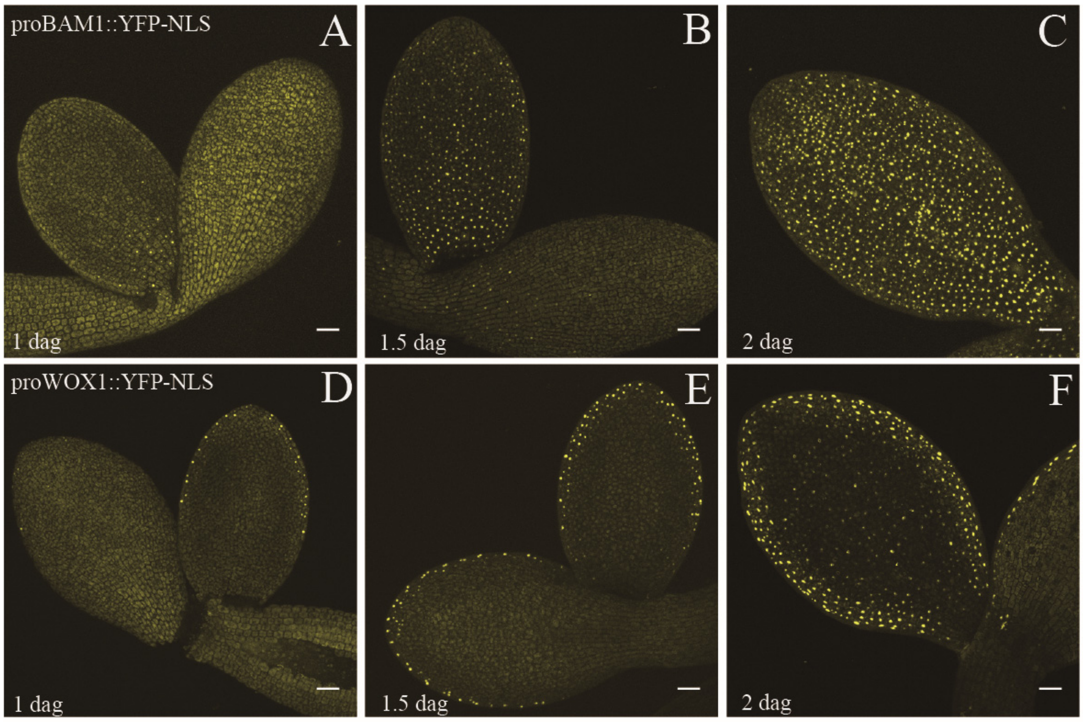

Figure S2 BAM1 and WOX1 expression pattern in young cotyledon

(A-C) proBAM1::YFP-NLS expression pattern in Col cotyledons at different stages. From left to right, 1 dag, 1.5 dag, 2 dag. Bars = 50 μm. YFP, yellow fluorescence protein. NLS, nuclear location sequence.

(D-F) proWOX1::YFP-NLS expression pattern in Col cotyledons at different stages. From left to right, 1 dag, 1.5 dag, 2 dag. Bars = 50 μm.

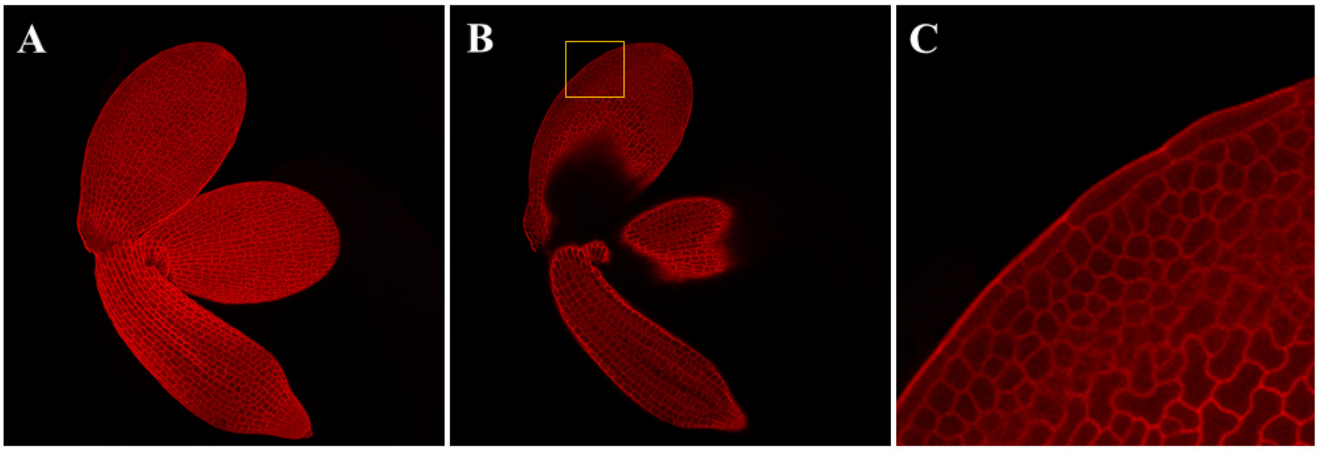

Figure S3 mPS-PI stained mature embryo of *bam1bam2bam3*

(A) .Maximum projection image of Z-stack of *bam1 bam2 bam3* mutant in embryo stage.

(B) Single slice of optimal section of margin cells of A.

(C) .Magnified image of B (outlined in yellow box)

Table S1 The sequence of primers used in genotyping and qRT-PCR

Primer name	Sequence information
BAM-RT-F	GCTAAATTCCTTCAAGATTCCGGTA
BAM-RT-R	GCACTATATCGACACCGTCTCCAAA
BAM1-F	AACTTTCTCTTTGGTTCAATCCCTG
BAM1-R	GCAATCGAACAAACAAGTAGTCCAA
Lab1.3	ATTTTGCCGATTTCGGAAC
UBQ10-F	AGATCCAGGACAAGGAGGTATTC
UBQ10-R	CGCAGGACCAAGTGAAGAGTAG
B1-proWOX1-F	GGGGACAAGTTTGTACAAAAAAGCAGGCTCA TTGGGCCAAAGTCTGATTTATATAA
B2-ProWOX1-R	GGGGACCACTTTGTACAAGAAAGCTGGGTC TTTGGTGTGTACTTAATTTATATGT
B1-proBAM1-F	GGGGACAAGTTTGTACAAAAAAGCAGGCTCA ATGATCCGATCCTCAAAAGTATGTA

B2-proBAM1-R	GGGGACCACTTTGTACAAGAAAGCTGGGTC TGTTTCTCTCTATCTCTCTTGTGTG
LB-1	AAGAAAATGCCGATACTTCATTGGC
WOX1-F	CTTTCCAGTCTCTCTTTCCCTTGTT
WOX1-R	AGATATATACCTCTGGTTGCGTGTC
PRS-F	ACGTAATGTAATAATTTTGGGAGCA
PRS-R	TTTGGTGCAGTAATATTCATTCATC
